# Characterizing Cellular Differentiation Potency and Waddington Landscape via Energy Indicator

**DOI:** 10.34133/research.0118

**Published:** 2023-04-11

**Authors:** Hanshuang Li, Chunshen Long, Yan Hong, Liaofu Luo, Yongchun Zuo

**Affiliations:** ^1^ State Key Laboratory of Reproductive Regulation and Breeding of Grassland Livestock, College of Life Sciences, Inner Mongolia University, Hohhot 010070, China.; ^2^ Department of Physics, Inner Mongolia University, Hohhot 010070, China.

## Abstract

The precise characterization of cellular differentiation potency remains an open question, which is fundamentally important for deciphering the dynamics mechanism related to cell fate transition. We quantitatively evaluated the differentiation potency of different stem cells based on the Hopfield neural network (HNN). The results emphasized that cellular differentiation potency can be approximated by Hopfield energy values. We then profiled the Waddington energy landscape of embryogenesis and cell reprogramming processes. The energy landscape at single-cell resolution further confirmed that cell fate decision is progressively specified in a continuous process. Moreover, the transition of cells from one steady state to another in embryogenesis and cell reprogramming processes was dynamically simulated on the energy ladder. These two processes can be metaphorized as the motion of descending and ascending ladders, respectively. We further deciphered the dynamics of the gene regulatory network (GRN) for driving cell fate transition. Our study proposes a new energy indicator to quantitatively characterize cellular differentiation potency without prior knowledge, facilitating the further exploration of the potential mechanism of cellular plasticity.

## Introduction

The establishment of cells with high differentiation potentials is fundamental to the production of mature cell types efficiently, which provides a new opportunity for regenerative medicine studies and therapeutic applications. Recently, a variety of stem cells with different differentiation potentials, including 2-cell like cells (2CLCs) [1–3], 8-cell like cells (8CLCs) [4], totipotent-like stem cells (TLSCs) [5], totipotent blastomere-like cells (TBLCs) [6], and extended pluripotent stem cells (EPSCs) [7], have also been captured under different culture conditions [8]. Cells are located at various locations in the Waddington landscape, corresponding to multiple differentiation potentials [9,10]. During the embryogenesis process, cells undergo a series of different states, from the initial totipotent or pluripotent state to the final lineage commitment state. With the discovery of Yamanaka factors [11], differentiated somatic cells can be reprogrammed from the bottom of Waddington's landscape back to the pluripotent state, which allows a cell's fate to be redirected [12,13].

Conventionally, characterizing cellular differentiation potency relies on transcriptomic signatures of known markers [14]. For example, a hallmark of pluripotent cells is the expression of pluripotent genes such as *Pou5f1*, *Zfp42*, and *Nanog* [15]. The totipotent cells are ascertained by the expression of totipotency molecular features such as *Dux*, the *Zscan4* gene cluster, and *MERVL* [16]. However, it is not sufficient to provide a new proxy for the Waddington landscape to quantitatively describe the differentiation potency of cells based on just a few markers [17,18]. Traditional marker-based methods are also not suitable for identifying novel cell populations [18]. It seems that the establishment of computational models is an important issue for describing the cellular plasticity and enhancing our understanding of cell fate conversions [19]. For instance, the SLICE model based on Shannon entropy was established to quantitatively measure cellular differentiation states and reconstruct cell differentiation lineages [18]. Then, a general algorithm called SCENT was developed to estimate cellular plasticity in terms of signaling entropy [17]. Another proposed model, called Markov chain entropy (MCE), quantifies cell differentiation potency by integrating the single-cell RNA sequencing (scRNA-seq) profile of a cell with a protein–protein interaction (PPI) network [20]. Moreover, Schiebinger et al. [21] described a conceptual framework, implemented in a method called Waddington-OT, to reconstruct the Waddington landscape in the reprogramming process and identify the important transcription factors (TFs) that regulate cell fates. Typically, TFs cooperate with other factors constructing a gene regulatory network (GRN) to drive the transformation of cells from one state to another [22,23]. These mathematical frameworks serve as an effective approach to understand cellular identity maintenance and fate transition.

Hopfield neural network (HNN) can recall a host of memorized patterns from input patterns associated with distinct attractors of the network [24,25]. Due to its prescriptive design, HNN has been applied in a variety of fields including associative memory [26], characterizing cancer subtypes [27], investigation of cell cycle dynamics [28,29], and construction of the epigenetic landscape [30,31]. But it is still a challenge to quantitatively characterize cellular differentiation potency and Waddington landscape. Here, HNN was used to quantitatively evaluate the differentiation potency of different stem cells based on highly variable genes (HVGs). The results showed that the cellular differentiation potency can be approximated by the Hopfield energy. Next, we simulated Waddington's energy landscape during the embryogenesis and reprogramming process containing explicit time information. The landscape elevation can be defined by Hopfield energy values in both processes, and the energy values correlated well to the differentiation potentials of the corresponding cell state. Furthermore, the robustness of Hopfield energy in the above evaluation was also discussed through the network random disturbance. Ultimately, the dynamics of core GRN that drives cell fate transition was also identified, which provides an effective strategy for purposefully controlling the cell fate transition.

## Results

### Hopfield energy evaluates the differentiation potency of different stem cells

**Fig. 1.**
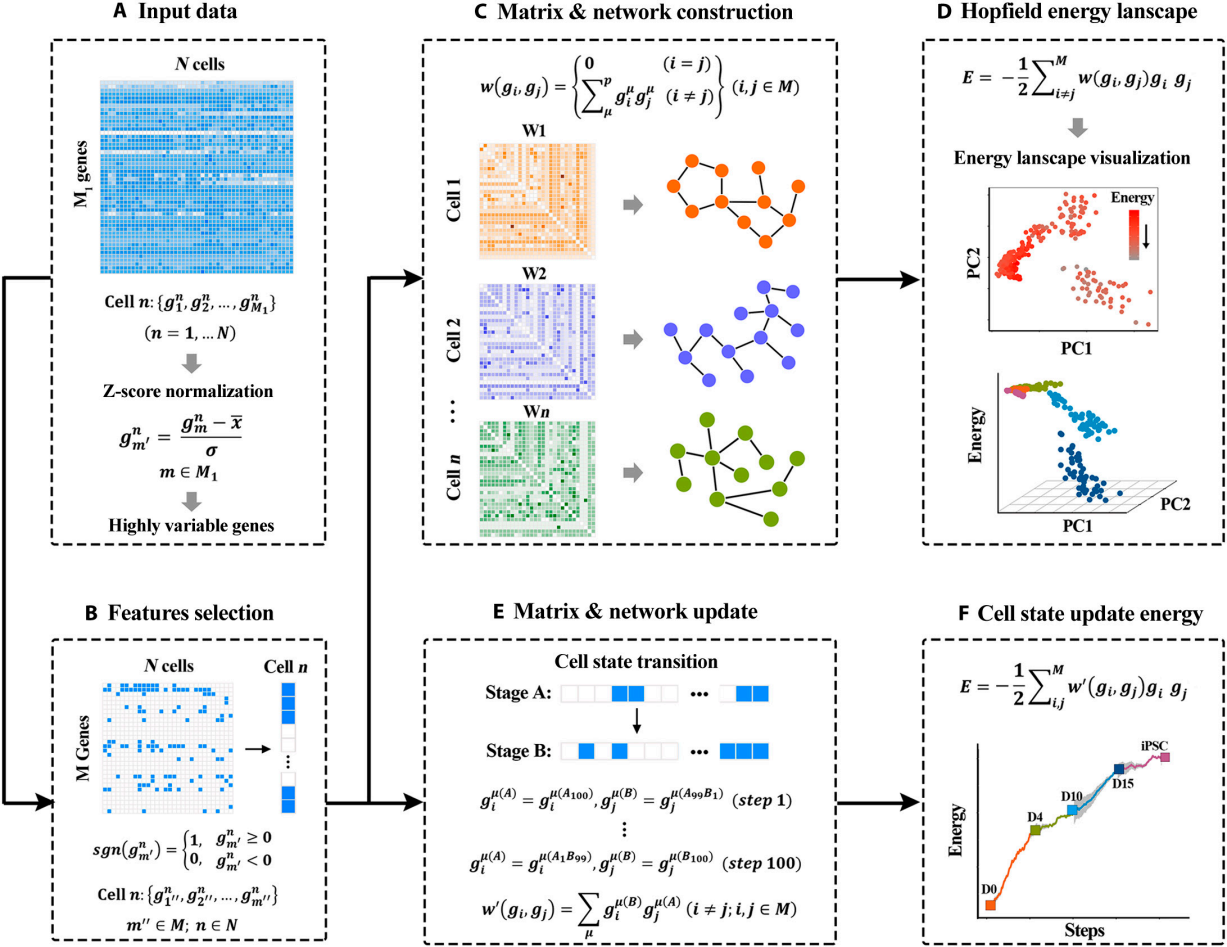
(A to F) The overall schematic flow of the Waddington's energy landscape (see Materials and Methods for detailed description).

**Fig. 2.**
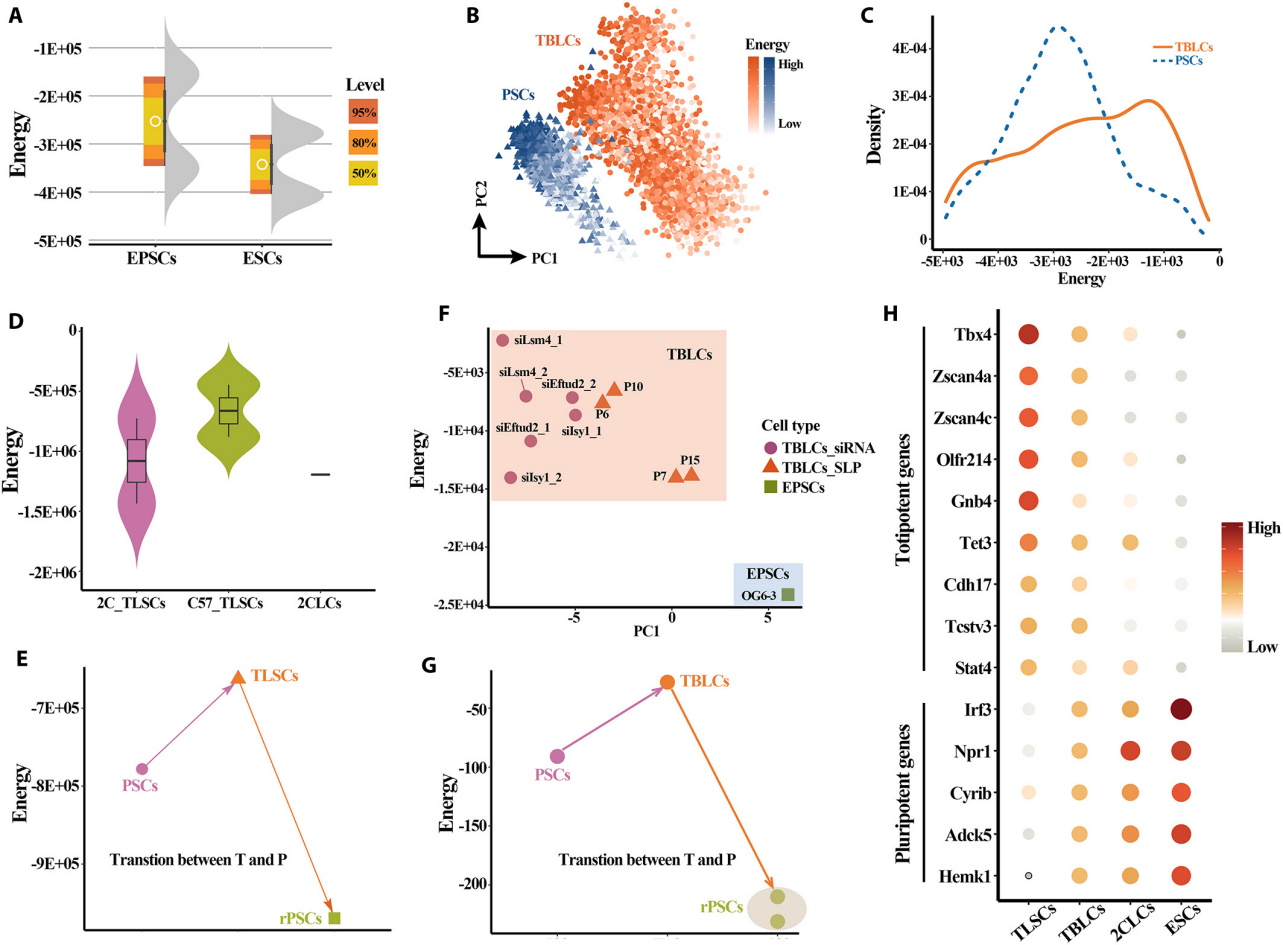
Hopfield energy measures the differentiation potential of stem cells. (A) Hopfield energy indicating the differentiation potential of EPSCs and ESCs. The legend shows the confidence level. (B) PCA of transcriptome in mouse TBLCs and PSCs. The *x* and *y* axes represent the first and second principal components of the data; the color represents the energy values of cells. (C) Density distribution of Hopfield energy in TBLCs and PSCs. (D) Violin plots showing the difference of Hopfield energy between TLSCs and 2CLCs. 2C_TLSCs and C57_TLSCs represent the TLSCs derived from mouse embryos and ESCs, respectively. (E) Change of Hopfield energy with the transition of cell state between pluripotent (P) and totipotent (T). The data were reanalyzed and derived from Yang et al. [5]. (F) The distribution of energy levels in mouse TBLCs (siRNA), TBLCs [serum/LIF/pladienolide B medium (SLP)], and EPSCs. The *x* axis represents the first principal components (PC1) of the data; the *y* axis represents the energy values of the corresponding cells. (G) change of Hopfield energy with the transition of cell state between pluripotent (P) and totipotent (T). The data were reanalyzed and derived from Shen et al. [6]. (H) Dotplots of expression of totipotent and pluripotency genes in TLSCs [5], TBLCs [6], 2CLCs [5], and ESCs [7].

Estimating the differentiation potency of cells is one of the important tasks for dissecting the diversity of cellular functions. With the deepening of research, a variety of stem cells with different differentiation potentials have been characterized, including TLSCs [5], TBLCs [6], EPSCs [7], and 2CLCs [5]. Traditionally, the differentiation potency of these stem cells is determined by the expression of known totipotent or pluripotent markers [14]. Especially, there are still certain technical obstacles for identifying these stem cells. To address this conundrum, we attempted to quantitatively assess the differentiation potency of these stem cells from different sources using Hopfield energy (Fig. 1A to D). Confirming our hypothesis, EPSCs exhibited higher energy values than embryonic stem cells (ESCs) (Fig. 2A), consistent with their known high differentiation potency [7]. Moreover, the transcriptome-based principal components analysis (PCA) clustering analysis proved that TBLCs and pluripotent stem cells (PSCs) followed different transcriptome dynamics (Fig. 2B). Likewise, TBLCs were assigned obviously higher energy than PSCs (Fig. 2B and C), suggesting that TBLCs attained higher differentiation potency than PSCs. This result was supported by the mouse chimeric assays that TBLCs have a robust capability to generate multiple embryonic and extraembryonic cell lineages [6]. We also calculated the energy values of TLSCs and 2CLCs to unequivocally compare the differentiation potency of these 2 types of stem cells (Fig. 2D). The highest energy values were attained by TLSCs (Fig. 2D), which is consistent with that TLSCs have high cell plasticity [5]. Next, we tested whether energy values could depict the transition of cells between pluripotent and totipotent states. The result showed that as PSCs progressed to TLSCs, cells had increased energy levels. Conversely, with the withdrawal of totipotency, the energy value of cells decreases (Fig. 2E). Consistent with Hopfield energy being an indicator of cell plasticity, we also observed that TBLCs [transiently induced by small interfering RNAs (siRNAs) or cultured in serum/leukemia inhibitory factor (LIF)/pladienolide B medium] exhibited obviously higher energy values than EPSCs (Fig. 2F). Crucially, the dynamic transition between PSCs and TBLCs can be quantitatively characterized by Hopfield energy values (Fig. 2G), showing that Hopfield energy provides a more general measure of differentiation potency than pluripotent signatures.

Next, we compared the molecular features of ESCs to that of totipotent cell types, including TLSCs, TBLCs, and 2CLCs. Compared with ESCs, most totipotent genes induced particularly in 2-cell embryos were broadly activated in TLSCs, TBLCs, and 2CLCs, consistent with the fact that these 3 types of stem cells were like 2-cell embryos. Especially, TLSCs were obviously enriched with the markers of 2-cell embryos, such as *Zscan4a*, *Zscan4c*, and *Tcstv3*, which were undetectable in ESCs (Fig. 2H). Contrary to this, ESCs highly expressed pluripotent genes, such as *Irf3*, *Npr1*, *Cyrib*, and *Adck5*, further confirming that ESCs were enriched with pluripotent genes, whereas the other 3 types of stem cells were enriched with totipotent genes (Fig. 2H). These above results indicate that Hopfield energy can approximate the differentiation potential of the cell without prior knowledge, in which high Hopfield energy represents high differentiation potency and low energy indicates low differentiation potency.

### Quantifying Waddington's landscape using Hopfield energy

**Fig. 3.**
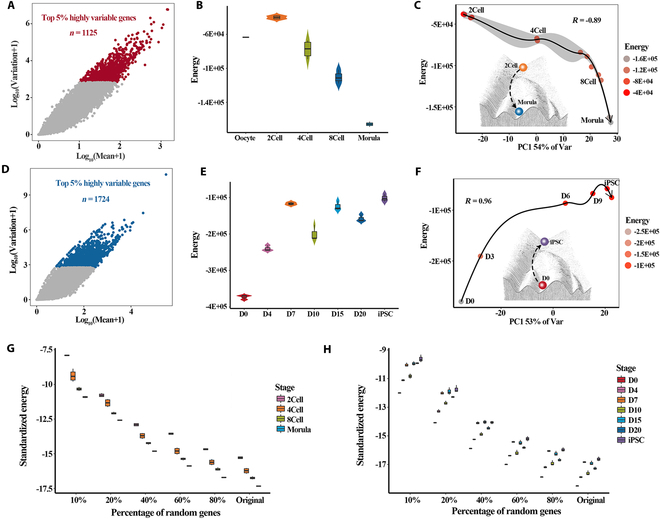
Hopfield energy simulates embryogenesis and cell reprogramming processes and perturbation analysis of network. (A) Screening of top 5% HVGs in the pre-implantation embryo development process. (B) Hopfield energy at various stages of embryo development. (C) Fitting model of embryogenesis inferred by Waddington's energy landscape. (D) Screening of top 5% HVGs in the somatic reprogramming process. (E) Hopfield energy at various stages of the reprogramming process. (F) Fitting model of cell reprogramming is inferred by Waddington's energy landscape. (G and H) Perturbation analysis of Hopfield network. The *x* axis represents original genes and 10, 20, 40, 60, and 80% of randomly selected genes from original genes. The *y* axis represents the standardized energy values of networks generated from these genes in embryogenesis (G) and reprogramming process (H), respectively.

Given the potential relationship between Hopfield energy and cell differentiation potential, we focused on 2 representative biological processes with explicit time information: the embryogenesis process and the reprogramming process. As described in Materials and Methods (Fig. 1A to D), we individually screened the top 5% of HVGs within these 2 processes and constructed a Hopfield network constituted with these HVGs in each cell (Fig. 3A and D). Based on these HVGs, we further calculated the Hopfield energy of cells at each stage for these 2 biological processes. The results showed that 2-cell embryos with high differentiation potential had the highest energy value, followed by 4-cell and 8-cell embryos, while embryos in the morula stage attained the lowest value (Fig. 3B). During the reprogramming process, the mouse embryo fibroblasts (MEFs) exhibited lower energy values than induced pluripotent stem cells (iPSCs). The energy of cells in the intermediate state of reprogramming was between MEFs and iPSCs (Fig. 3E).

Next, we simulated the dynamic landscapes of Hopfield energy during embryogenesis and cell reprogramming process, respectively. Correspondingly, the energy levels of cells exhibit a consistent decrease with the actual development route. Of note, the principal component (PC) 1 axis coincided with the developmental path (*R* = −0.89) (Fig. 3C). Briefly, with the decrease of the Waddington landscape elevation where the cells are located, corresponding energy levels of the cells also decrease gradually (Fig. 3C). Subsequently, we applied the above analysis to the reprogramming process. The results showed that the transcriptome dynamics in the reprogramming process followed 3 stages: early reprogramming (MEFs, D3), intermediate reprogramming (D6, D9), and last reprogramming (D12, iPSCs). For cells in the early reprogramming stages, we observed relatively low energy values. On induction, these cells enter an intermediate state, where we observed an increase in energy to D6_Energy_ = −8.63E+04 followed by D9_Energy_ = −6.72E+04 before the cells reached the pluripotent state (iPSCs) with the highest energy iPSCs_Energy_ = −5.76E+04 (Fig. 3F). The result showed that the cells progressively acquire higher energy values with the withdrawal of fibroblast differentiation degree and the gradual establishment of pluripotency (*R* = 0.96) (Fig. 3F), which is contrary to the decline of energy in the development process.

To further test the robustness of Hopfield energy in quantifying Waddington's landscape, we progressively perturbed gene expression values with different ratios and calculated the energy of cells at each given stage (Fig. 3G and H). The results meant that when 20% of the gene expression levels were perturbed, in either embryogenesis or reprogramming process, the energy values of cells at the different stages were close to the original network. Most importantly, even if the disturbance levels were as high as 90%, the change trend of energy of cells at each stage remained stable in the whole biological process, showing that Hopfield energy is robust to quantifying Waddington's landscape. Altogether, these results demonstrated that the height of the landscape was highly related to the differentiation potential of the corresponding cell state, which can be estimated by Hopfield energy. Notably, whether it is embryogenesis or reprogramming process, the dynamic transformation of cell fate on Waddington's landscape can be quantitatively described by Hopfield energy.

### Modeling cellular identity transition with Hopfield energy at single-cell resolution

**Fig. 4.**
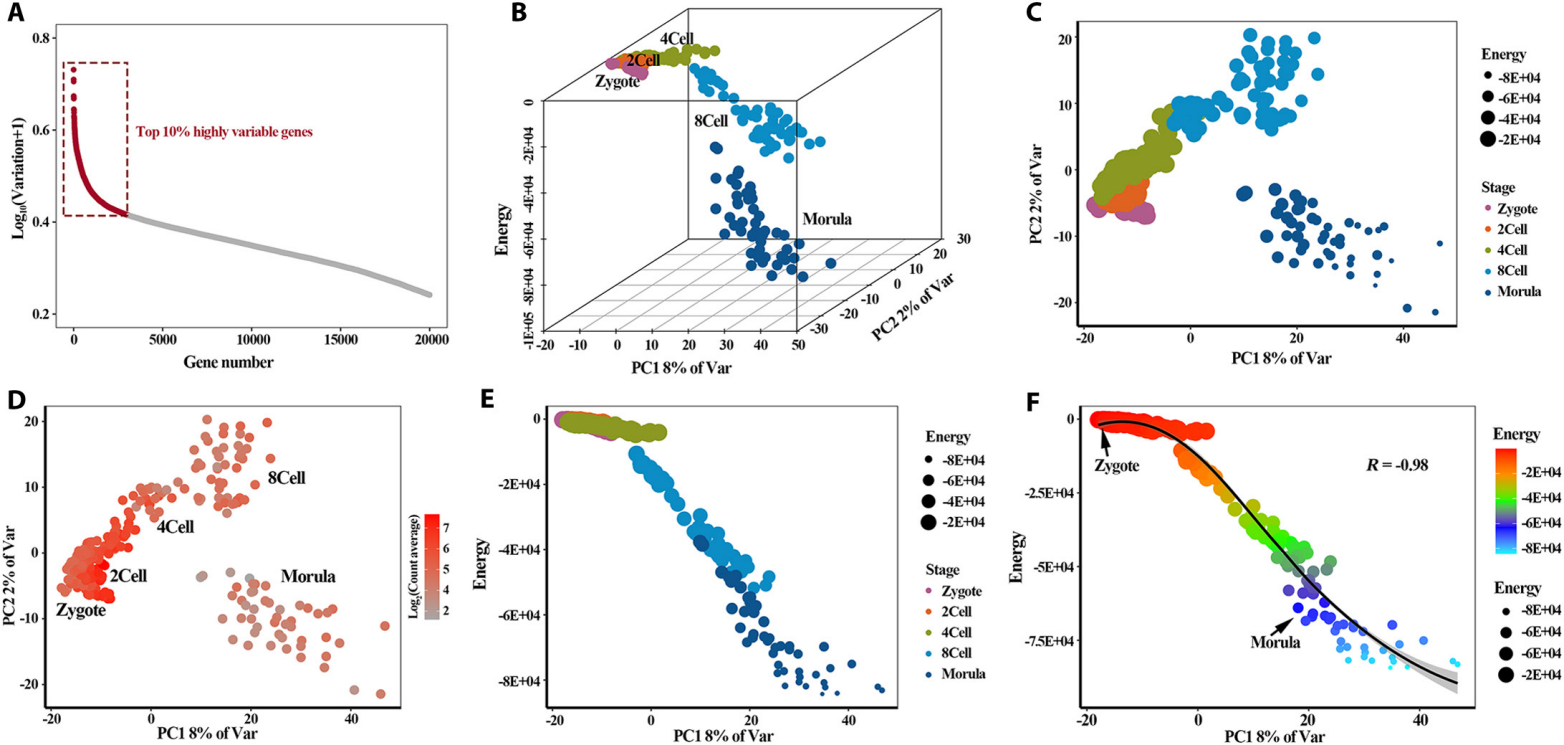
Constructing Waddington' energy landscape of embryogenesis based on scRNA-seq data. (A) Screening of top 10% HVGs in the pre-implantation embryo development process. (B) Hopfield energy landscape of embryo development. The *x* and *y* axes represent the first and second principal components of the data; the *z* axis represents the energy values of cells with different development stages. (C) PCA plot of the Zscan family activities and the point size indicates the energy value of each cell. (D) PCA plot of the Zscan family activities colored by the average expression levels of genes. (E) Scatterplot showing the dynamics of energy levels in the development process. The decrease of energy of embryos correlates with the progression of development. (F) Scatterplot showing the fitting model of embryogenesis process inferred by Hopfield energy.

Cell fate decision has been considered as a continuous process at a single-cell level rather than a discrete hierarchical process [32], but the bulk RNA sequencing (RNA-seq) tends to mask infrequent molecular events that are essential for cellular transitions [33]. The advance of scRNA-seq provides a possibility to simulate the process of cell fate decision using a mathematical model. However, the traditional marker-based methods are difficult to comprehensively summarize the pluripotency differences of the intermediates of cell fate decision. To validate whether our method is also applicable and robust to scRNA-seq data with explicit time information, we further applied this method to the scRNA-seq dataset with known developmental time information. The data included zygotes, 2-cell embryos, 4-cell embryos, 8-cell embryos, and morula. First, we screened out the top 10% HVGs in the whole embryogenesis process to construct the GRN in each cell (Fig. 4A) and then calculated the Hopfield energy of these cells. Based on the PCA, the dynamic transition of these cells during embryogenesis was depicted (Fig. 4B). The result indicated that the cells at the same stage followed similar transcriptome dynamics. Furthermore, we described the dynamics of Hopfield energy during embryogenesis, showing that the energy values of cells decreased with a gradual decline of landscape elevations in 3-dimensional space (Fig. 4B). Consistently, when we projected the energy values into the size of each cell (Fig. 4C), the results revealed that starting from the zygote stage, with the descent of cell differentiation potential, the energy of cells gradually reduced during the embryogenesis process.

Further, we observed that the *Zscan* family was activated in the 2-cell stage of embryogenesis and continued until the 8-cell stage (Fig. 4D), consistent with that these factors play a mechanistic role in mouse zygotic genome activation (ZGA) [34,35]. Expectedly, the PC1 axis overlapped the development path to some extent (Fig. 4E), and the energy values of the cells along the PC1 axis were gradually decreasing. To further explore whether Hopfield energy and first principal components (PC1) can simulate the process of cell development, we constructed the energy fitting curve of cells in embryogenesis (*R* = −0.98) (Fig. 4F). The process of embryonic development follows a gradual reduction in energy, further confirming that cell fate decision is progressively specified in a continuous transcriptional landscape [32].

### The dynamic landscape of Waddington's energy

**Fig. 5.**
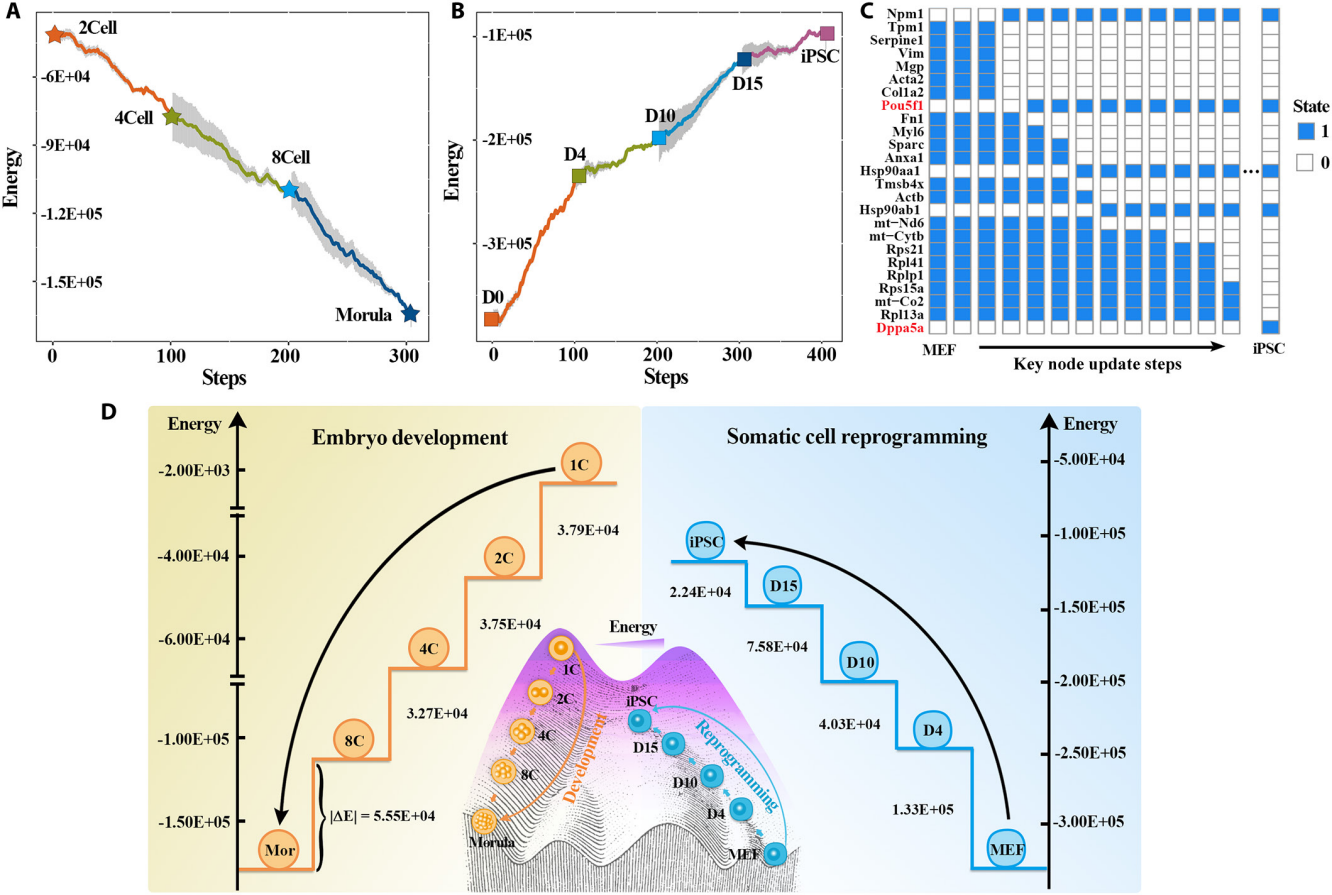
The reconstruction of Waddington's energy landscape and the dynamic transition of GRNs. (A and B) Line plots showing the dynamics of energy values of the cell state transition during the development process (A) and reprogramming process (B), respectively. (C) Dynamic update of key nodes in Hopfield networks during induction from MEFs to iPSCs. The frequency of key updates (steps) is shown in the figure. (D) Schematic diagram of Waddington's energy landscape in embryogenesis (left) and cell reprogramming (right). The value next to the ladder represents absolute values of the energy differences (|∆*E*|) between the 2 stages.

The transition of cell state is a dynamic and continuous process, but how to intuitively describe the intermediate process of cell state transformation and precisely identify the driving factors in the whole process remains poorly understood. To further gain biological insight into the transition of cell states in embryogenesis, we took 2-cell stage as the initial state (old balance) and 4-cell stage as the new balance and reconstruct the energy landscape of this process using the Hopfield network update principle as described in Materials and Methods (Fig. 1E and F). As expected, the result showed that the embryos can break the old balance (i.e., 2-cell state) and move toward a new balance (i.e., 4-cell state) and the state renewal from 4-cell to 8-cell and the 8-cell to morula state were similar to the above results (Fig. 5A), confirming that the reconstructed Waddington's energy landscape can reflect the progression of embryo development. Moreover, we also observed that there were differences in the energy required for the transformation between cell states at different stages. Therefore, whether the possibility of cell fate can be inferred from the energy difference between cell states needs to be further explored.

During the reprogramming process, MEFs will break the current state (D0) and undergo a series of stable states (D4, D10, D15), finally reaching a new balance state (iPSCs) (Fig. 5B). In this process, with the establishment of cell pluripotency, the differentiated cells jump out of the potential well like particles. Moreover, the GRN was also dynamically shifting during the induction of MEFs to iPSCs (Fig. 5C). Among them, known pluripotency genes including *Pou5f1* and *Dppa5a* are gradually activated, supporting that they function as drivers for facilitating reprogramming [33,36–38]. Meanwhile, we also deciphered some new factors (e.g., *Npm1*, *Hsp90ab1*, and *Hsp90aa1*) based on importance score (Table S1; see Materials and Methods). The activation of these factors is important in driving the network toward the iPSCs, which can provide new insights for experimental biologists to further explore the regulatory mechanisms of cell pluripotency. In all, the Hopfield network can not only reconstruct Waddington's energy landscape to simulate the state transition from one cell fate to another but also identify the dynamics of an important GRN to drive this process.

## Discussion

Waddington’s landscape as a “colorful metaphor” is often used to describe the transition of cell fates [39,40]. To model this landscape, Huang [41] connected the landscape elevation to the likelihood of the corresponding cell state based on “quasi-potential”. Subsequently, numerous popular “entropy” models were introduced to infer landscapes from biological data [17,18,20,42]. For example, SLICE introduced Shannon entropy to measure the differentiation potential of cells by characterizing the multiple potentials or uncertainty of a biological system. In our study, Hopfield energy is defined according to the relationship among genes and reflects the stability of the network.

In our study, we quantitatively evaluated and compared the differentiation potential of different stem cells and then profiled Waddington's energy landscape in embryogenesis and cell reprogramming based on HNN. At the same time, cells with the same developmental stage or reprogramming stage have similar energy levels and can be gathered at the same height of the Waddington landscape. During embryogenesis, zygote with higher developmental potential has the highest energy value, while cells in the morula stage have lower energy (Fig. 5D). Similarly, in the process of reprogramming, iPSCs with high developmental potential have the highest energy, while differentiated fibroblasts attained the lowest energy (Fig. 5D). These results further determined that the differentiation potential of cells is related to the landscape elevation, which can be defined by energy values.

In addition, we simulated the dynamic transition of cell identity in these 2 biological processes on Hopfield energy ladder. The results suggested that the development process and reprogramming process can be metaphorized as the descending and ascending ladders, respectively. During embryogenesis, with the decrease of zygote-related components, the cells will experience a series of steady states, including 2-cells, 4-cells, and 8-cells, and finally reach the morula state (Fig. 5D). Conversely, during the fate transition from MEFs to iPSCs, the energy of cell gradually increases, consistent with the fact that reprogramming is a process of increasing versatility (Fig. 5D). This process is like the step-by-step jump of particles from a potential well, which is accompanied by the dynamic activation of the pluripotency regulatory network.

Notably, Hopfield energy is a scalar value associated with each state of a cell at a given time that is not directly related to physical form of energy. Hopfield energy is defined according to the relationship (connection) among genes and reflects the stability of the GRNs. This is the very reason why the Hopfield energy is an excellent proxy to quantitatively describe the cell plasticity characterized by the GRNs. Our study is an effort to move the qualitative Waddington landscape toward a quantitative model, which provides a general measure of differentiation potency independent of external knowledge. An extension of the Hopfield energy application is to quantify cell heterogeneity from the same populations, which allows for the potential discovery of putative cell types [43,44]. Additionally, the bias of cell fate determination is the result of multilayered collaborative regulation of several epigenetic modifications. Therefore, we hope to extend our model to other omics data and improve the Hopfield energy from a scalar indicator to a vector indicator to predict cell fate in the future work.

## Materials and Methods

### Data collection

Both the single-cell and bulk RNA-seq datasets analyzed in this study were downloaded from Gene Expression Omnibus (GEO) database. The detailed description of these data is as follows:

The accession number of TLSCs and 2CLCs is GSE185030 [5]. Both the single-cell and bulk RNA-seq datasets of TBLCs and PSCs were from GSE168728 [6]. The datasets of EPSCs and ESCs were downloaded from GSE80732 [7]. The 2 datasets related to embryogenesis were derived from Wang C et al. [45] and Wang Y et al. [46], respectively. The accession number of Wang C et al. dataset is GSE98150, which includes different embryos from MII oocyte to morula development stages. In Wang Y et al. dataset (GSE136714), a total of 280 single cells from different stages of embryonic development were distributed as follows: zygote (*n* = 26), 2-cell embryos (*n* = 48), 4-cell embryos (*n* = 94), 8-cell embryos (*n* = 64), and morula (*n* = 48). Moreover, the other 2 datasets related to cell reprogramming were derived from Cieply B et al. (GSE70022) [47] and Fang HT et al. (GSE99548) [48], respectively. These 2 datasets include different stages about MEFs reprogramming to the iPSCs.

### Bulk RNA-seq and single-cell RNA-seq data preprocessing

The bulk RNA-seq reads were trimmed by Trimmomatic (version 0.38) [49] and mapped to mm10 reference genome using Hisat2 (version 2.1.0) aligner for stranded reads with default parameters [50,51]. The retained reads were subsequently assembled by using Stringtie (version 1.3.5) [52]. Expression levels for genes were quantified to normalized FPKM (fragments per kilobase of exon model per million mapped reads) [35].

The scRNA-seq raw reads were trimmed with Trimmomatic (version 0.38) [49] to remove the primer sequences and low-quality bases. The trimmed reads were aligned to the mm10 genome with Hisat2 (version 2.1.0) with the default settings [50]. The reads mapped to each gene were counted based on HTseq (version 0.11.0) with default parameters [53].

### Input data preprocessing and feature selection

In this study, we first input the *N* × *M*_1_ dimensional matrix, where *N* is the set of input cells (for scRNA-seq) or samples (for bulk RNA-seq) and *M*_1_ is the gene set in these cells or samples (Fig. 1A).

$$\text{Cell}\;n:\{g_{1}^{n},g_{2}^{n},\ldots ,g_{M_{1}}^{n}\;\},(n=1,\ldots N)$$ (1)

Then, Z-score normalization [54] is performed on the data, as shown below:

$$g_{m^{\prime }}^{n}=\frac{g_{m}^{n}-\bar{x}}{\sigma },\left(m\in M_{1},\;-1\leq g_{m^{\prime }}^{n}\leq 1\right)$$ (2)

where $g_{m}^{n}$ is the expression level of gene *m* in cell *n*, and $\bar{x}$ and *σ* are the average expression level and standard deviation of the expression level of a given gene *m* in all cells, respectively. Then, by screening the top 5% or 10% HVGs from the processed data, we obtain a *N* × *M* matrix, where *M* is the gene set composed of HVGs in *N* cells (Fig. 1A).

The developmental state or reprogramming state of cells depends on a network composed of multiple genes as nodes. In our study, there are *M* genes corresponding to the input cell *n*. In order to be consistent with Hopfield network, we discretize the state of each gene into 2 possible values (0 or 1). That is, we use $\mathrm{sgn}(g_{m^{\prime }}^{n})$

$$g_{m^{\prime \prime }}^{n}=\mathrm{sgn}\left(g_{m^{\prime }}^{n}\right)=\left\{\begin{array}{c} 1,\\ 0, \end{array}\;\begin{array}{c} g_{m^{\prime }}^{n}\geq 0\\ g_{m^{\prime }}^{n}<0 \end{array},\;\left(m^{\prime \prime }\in M,n\in N\right)\right.$$ (3)

to define the state of a given gene *m*. Thus, the vector $\left\{g_{1^{\prime \prime }}^{n},g_{2^{\prime \prime }}^{n},\ldots ,g_{m^{\prime \prime }}^{n}\right\}$ composed of multiple genes with different state represents the state of cell *n* (Fig. 1B).

### Construction of Hopfield network at stable fixed point

HNN is a single-layer fully connected feedback neural network, which consists of nodes and weighted undirected edges [26]. The output of each node in the network is deduced from the interaction between this node and the input of other nodes. The interaction between *i*th and *j*th nodes is called connection *w_ij_* and {*w_ij_*} constitutes a weight matrix [31,55]. One of the fascinating aspects of the HNN is, like the other neural network, its function as an associate memory with a surprising fault tolerance with respect to both input data errors and internal failures. This fault tolerance is important for the biological application of the model.

In our study, the nodes of network can be regarded as genes and one can assume the connection *w*(*g_i_*, *g_j_*) between any pair of nodes (*g_i_*, *g_j_*) given by

wgigj=0i=j∑μpgiμgjμi≠jij∈M (4)

where $g_{i}^{\mu }\;$and $g_{j}^{\mu }$ represent the status of gene *i* and *j* in each cell or sample, respectively. In our study, *μ* denotes the state of a given cell or sample, and *p* is the number of stable states. When calculating the energy of a sample (or cell), the stable state refers to the current state of the sample (or cell). That is, cells or samples with clear phenotypic information are directly modeled as attractors (stable states) of the HNN. For single-cell data, *p* is taken as 1; for bulk RNA-seq data, *p* represents the number of samples with the same phenotype. When the network is at a stable fixed point, the connection *w*(*g_i_*, *g_j_*) is given using Eq. 4, and the connection is symmetric, *w*(*g_i_*, *g_j_*) = *w*(*g_j_*, *g_i_*). Thus, the zero diagonal symmetric weight matrix *W* [with matrix element *W_ij_* = *w*(*g_i_*, *g_j_*)] is obtained (as shown in Fig. 1C).

In Hopfield network theory, it is demonstrated that the fixed point is exactly the minimum of the energy function *E* (called Hopfield energy).

$$E=-\frac{1}{2}\sum_{i\neq j}^{M}w\left(g_{i},g_{j}\right)g_{i}g_{j}$$ (5)

Therefore, in our study, the fixed point of the network corresponds to the stable stage of development and reprogramming process (Fig. 1D).

### Hopfield network updates from one state to another

When we discuss the actual biological process, what we discussed is the cell transitions from state *A* to state *B* (Fig. 1E), but not the stable status. How to generalize the Hopfield network method to this case? Evidently, the symmetrical connection is no longer applicable. We should use asymmetrical connection, *w*(*g_i_*, *g_j_*) ≠ *w*(*g_j_*, *g_i_*). That is, we should modify equation (Eq. 4) and introduce an updated asymmetrical parameter *w*′(*g_i_*, *g_j_*) as follows:

$$w^{\prime }\left(g_{i},g_{j}\right)=\sum_{\mu }g_{i}^{\mu \left(B\right)}g_{j}^{\mu \left(A\right)}\;\left(i\neq j;i,j\in M\right)$$ (6)

where $g_{i}^{\mu \left(B\right)}$ denotes the state of gene *i* in a new state *B* and $g_{j}^{\mu \left(A\right)}$ indicates the state of gene *j* in the old state *A*. The definition of *μ* is the same as Eq. 4.

The state transition of cells from *A* to *B* can also be regarded as the process of breaking old balance *A* and reaching a new state *B*. Therefore, we use Eq. 6 and insert it into the following Eq. 7 to discuss state transition:

$$g_{i}^{B}=\mathrm{sgn}\left(\sum_{i\neq j}w^{\prime }\left(g_{i},g_{j}\right)g_{j}^{A}\;\right)\;\left(i,j\in M\right)$$ (7)

Equation 7 is a set of cell automaton equation where $g_{i}^{B}$ is either “pulled toward” or “pushed away” from $g_{j}^{A}$ depending on the sign of *w*′(*g_i_*, *g_j_*).

In order to identify the core factors driving cell fate transition, a simple matching coefficient-based importance score (IS) was proposed. Theoretically, IS can measure the contribution of each gene's state change (node update) driving the network toward the defined stable state, and its definition was as follows:

$$\text{IS}=\frac{A_{i}^{\left(2\right)}\cap A_{i}^{\left(0\right)}-A_{i}^{\left(1\right)}\cap A_{i}^{\left(0\right)}}{A_{i}^{\left(0\right)}}\;$$ (8)

where $A_{i}^{\left(0\right)}$ represents the network connection of *i*th gene in stable state; $A_{i}^{\left(1\right)}$and $A_{i}^{\left(2\right)}$ represent the network connection of the *i*th gene before and after its state change. Network connections related to *i*th genes were defined as $A_{i}^{\left(\tau \right)}=\left\{a_{i1}^{\left(\tau \right)},a_{i2}^{\left(\tau \right)},\ldots ,a_{\mathrm{iM}}^{\left(\tau \right)}\right\},\tau \in \left\{0,1,2\right\}$, with $a_{ij}^{\left(\tau \right)}$ the connection state of the *i*th gene and *j*th gene. Here, $a_{ij}^{\left(\tau \right)}$ can be calculated by the difference between the binary expression value of these 2 genes. Genes with IS > 0.1 were retained.

### Hopfield energy model of the Waddington's landscape

In order to reconstruct the energy landscape of cells during the transition from state *A* to state *B*, we decompose the process into several steps, for example, 100 steps as follows:

giμA=giμA100,gjμB=gjμA99B1step1giμA=giμA99B1,gjμB=gjμA98B2step2giμA=giμA98B2,gjμB=gjμA97B3step3⋮giμA=giμA1B99,gjμB=gjμB100step100 (9)

Here, $g_{i}^{\mu \left(A_{100}\right)}$ represents that the state of gene *i* is taken from the initial state *A* and $g_{j}^{\mu \left(B_{100}\right)}$ shows that the state of gene *j* is taken from the final state *B*, while $g_{j}^{\mu \left(A_{99}B_{1}\right)}\;$ in step 1 means that state *A* contributes 99% and state *B* contributes 1%, that is, 99% nodes are randomly selected from the network in state *A*, and the remaining 1% nodes are selected from state *B* to form a new network, $g_{i}^{\mu \left(A_{1}B_{99}\right)}\;$ in step 100 means 1% nodes are randomly selected from the network of state *A* and the remaining 99% nodes are selected from state *B* to form a new network, and so on.

For each step, the connection between any pair of gene pairs is calculated by using Eq. 6 (Fig. 1E). Since the number of steps is large enough, the energy in each step of transition can be calculated approximately by using a formula like Eq. 10, that is:

$$E=-\frac{1}{2}\sum_{i,j}^{M}w^{\prime }(g_{i}g_{j})g_{i}g_{j}$$ (10)

Based on above steps, Hopfield energy can describe not only the steady states but also the transition between them. Therefore, Hopfield energy gives a quantitative and calculable approach to simulate the Waddington landscape. By assuming that the Hopfield energy correlated with the landscape elevation, the dynamic mechanism of the system's motion (cell state transition) on the Waddington landscape is given (Fig. 1F).

### PCA of gene expression patterns

PCA was conducted using prcomp function in R. In our study, we focus on principal components (PC) 1 and 2. The implementation and visualization of Waddington's energy landscape are performed using R (version 4.0.3; http://www.r-project.org).

## Data Availability

All sequencing data associated with this study were downloaded from GEO database. The accession number of TLSCs and 2CLCs is GSE185030 [5]. Both the single-cell and bulk RNA-seq datasets of TBLCs and PSCs were from GSE168728 [6]. The datasets of EPSCs and ESCs were obtained in GSE80732 [7]. The 2 datasets related to embryogenesis were derived from GEO under accession numbers GSE98150 [45] and GSE136714 [46], respectively. The other 2 datasets related to cell reprogramming were derived from GSE70022 [47] and GSE99548 [48], respectively. The source code of this study is freely available in the GitHub repository (https://github.com/lhshuang/HopfieldEnergy).
